# Automated cortical thickness measurement of the mandibular condyle head on CBCT images using a deep learning method

**DOI:** 10.1038/s41598-021-94362-7

**Published:** 2021-07-21

**Authors:** Young Hyun Kim, Jin Young Shin, Ari Lee, Seungtae Park, Sang-Sun Han, Hyung Ju Hwang

**Affiliations:** 1 Department of Oral and Maxillofacial Radiology, Yonsei University College of Dentistry, 50-1 Yonsei-ro Seodaemun-gu, Seoul, 03722 South Korea; 2 Department of Mathematics, Pohang University of Science and Technology, 150 Jigok-ro Nam-gu, Pohang-si, Gyeongsangbuk-do 37666 South Korea

**Keywords:** 3-D reconstruction, Computer science, Machine learning, Medical research

## Abstract

This study proposes a deep learning model for cortical bone segmentation in the mandibular condyle head using cone-beam computed tomography (CBCT) and an automated method for measuring cortical thickness with a color display based on the segmentation results. In total, 12,800 CBCT images from 25 normal subjects, manually labeled by an oral radiologist, served as the gold-standard. The segmentation model combined a modified U-Net and a convolutional neural network for target region classification. Model performance was evaluated using intersection over union (IoU) and the Hausdorff distance in comparison with the gold standard. The second automated model measured the cortical thickness based on a three-dimensional (3D) model rendered from the segmentation results and presented a color visualization of the measurements. The IoU and Hausdorff distance showed high accuracy (0.870 and 0.928 for marrow bone and 0.734 and 1.247 for cortical bone, respectively). A visual comparison of the 3D color maps showed a similar trend to the gold standard. This algorithm for automatic segmentation of the mandibular condyle head and visualization of the measured cortical thickness as a 3D-rendered model with a color map may contribute to the automated quantification of bone thickness changes of the temporomandibular joint complex on CBCT.

## Introduction

The temporomandibular joint (TMJ) complex is composed of bony components and the surrounding musculature^1^. The mandibular condyle rotates and translates anteriorly as the mouth opens. Simultaneously, the joint disc moves into position between the condyle and the articular eminence^2^. This process is often accompanied by abnormal dislocation of the joint disc, which is the most common finding of temporomandibular joint disorder (TMD)^3,4^ and results in degenerative bony changes on the mandibular condyle head. In the early stage, bony flattening or sclerosis is observed, and as the disease progresses, the cortical surface shows erosive bony changes, which are regarded as indicating osteoarthritis (OA)^5–7^. OA of the TMJ may cause pain, dysfunction, clicking sounds, and headache^1,7^. Hence, it is important to make an accurate diagnosis at the early stage of bony changes to ensure suitable treatment.

With the worldwide increase in TMJ-OA, cone-beam computed tomography (CBCT) is used to provide useful information on osseous components of the TMJ through high-resolution multidirectional (axial, coronal, and sagittal) reconstructed images^8–10^. Nevertheless, clinicians often find it difficult to reach consistent conclusions regarding bony changes in TMJ-OA based on CBCT image findings^11^. In qualitative evaluations of the condyle head, the diagnostic terminology for bony changes (e.g., mild, moderate, and severe) gives rise to subjective judgments by clinicians when interpreting images, making a quantitative diagnosis difficult^12^.

The shape correspondence approach has been applied for the quantitative assessment of condylar head changes^13,14^. Using this technique, a reference three-dimensional (3D) condyle head was generated and compared to the condylar model of patients with TMJ-OA, and significant differences were found in the morphology in the TMJ-OA group. The shape-based method is useful for diagnosing disease stages accompanied by morphological changes; however, early cortical bone changes (e.g., thinning or thickening) may be difficult to observe using this method. Meanwhile, Ahmad and Schiffman^12^ proposed two grades for degenerative joint disease based on a thickness of 2 mm of the cortical layer on the condyle head. However, the difficulties of selecting the appropriate diagnostic slices and measuring the cortical plate remain obstacles to a reliable evaluation by clinicians.

Various conventional methods such as thresholds^15,16^, a model-based approach^17^, an intensity clustering-based model^18^, and statistical shape models^19,20^ have been explored as ways to segment target anatomical structures based on medical images. These methods ensure accurate segmentation results when used by a well-trained expert, but some issues have been pointed out regarding the CBCT image-based condylar head segmentation task, such as the time-consuming nature of the task, difficulties in setting reliable threshold settings, and low reproducibility among researchers^21–24^. In addition, since the existing methods are mostly based on gray levels, it is difficult to obtain reliable segmentation results for CBCT images suffering from image non-uniformity and high image noise^24–27^.

Convolutional neural networks (CNNs) have emerged as a breakthrough approach with the potential to eliminate the aforementioned shortcomings of conventional methods through complete automation of the segmentation process^28,29^. In recent studies, researchers have proposed automatic segmentation and thickness estimation methods for various body parts, such as the brain, knee, tibia, and femur, based on magnetic resonance imaging, high-resolution computed tomography (CT), and micro-CT^15,17,18,30^. In comparison with other body parts, the mandibular condyle head is considered particularly difficult for obtaining accurate and reliable segmentation outcomes due to its surrounding complex anatomical structures and relatively low bone density^31^. The fact that the performance variability of deep learning models depends on the data source may be another reason to develop a CBCT-based condylar head segmentation model^28,29^. However, to the best of our knowledge, no CBCT-based studies have used deep learning approaches to target the mandibular condyle region for automatic segmentation of the marrow and cortical bones.

To propose a quantitative and reliable automated evaluation method, we developed a deep learning model for cortical bone segmentation of the mandibular condyle head on CBCT images, and a color display model that visualizes the automatically measured cortical thickness in 3D based on the segmentation results. First, the deep learning model includes a U-Net-based segmentation algorithm^32^ and a CNN-based classifier that determines whether a condyle head exists in each original image or not. This model is designed to automatically separate the cortical and marrow bones. To our knowledge, our study is the first proposal of a deep learning algorithm for automated segmentation of the cortical layer of the condyle using CBCT images. The second model stacks the segmented outputs of the first model and automatically measures the thickness along the surface area of the condyle head. The integrated model automatically estimates the cortical bone thickness of the segmented condyle head, and a color-mapped 3D-rendered image is provided to facilitate the straightforward diagnosis of bony changes.

We expect that the use of our proposed algorithm will make it easier for clinicians to obtain diagnostic information, which is our motivation for proposing this novel automated model for segmentation using a deep learning algorithm and color display program to represent automatically generated measurements of cortical thickness.

## Materials and methods

### Data preparation

This study was approved by the Institutional Review Board (IRB) of Yonsei Dental Hospital (No. 2-2019-0069) and was carried out in accordance with relevant guidelines and ethical regulations. Informed consent was waived by the IRB of Yonsei Dental Hospital because of the retrospective nature of the study. A total of 12,800 CBCT images from 25 anonymized subjects with no pathological bony changes on the mandibular condyle head were selected from the picture archiving and communication system at Yonsei University Dental Hospital. In each of the original images, the cortical bone and marrow bone of the condyle heads were manually annotated by an experienced oral and maxillofacial radiologist, and all labeled images were independently verified by another expert with over 20 years of experience. Labeling data with disagreements were corrected by consensus between the two oral radiologists.

All CBCT data was taken by an Alphard 3030 device (Asahi Roentgen Ind. Co., Ltd, Tokyo, Japan) at the department of oral and maxillofacial radiology. The acquired parameters were set as 80 kVp, 8 mA, an exposure time of 17 s, 15.4 × 15.4 cm^2^ field of view, and 0.3 mm voxel resolution. The mean age of the subjects was 36.0 years (range, 17–73 years), and there were 7 males and 18 females. To verify our algorithm, the CBCT data were divided into training, validation, and test sets as follows: 18 subjects in the training set (9216 slices), 5 subjects in the validation set (2560 slices), and 2 subjects in the test set (1024 slices). From each subject, 512 axial slices were obtained, and on average 9% of the axial slices contained the mandibular condyle head.

### Proposed segmentation model

**Figure 1:**
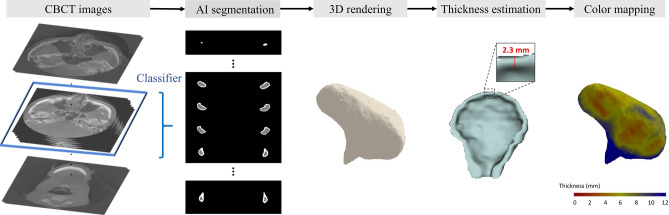
Overall process of the proposed algorithm.

Figure 1 displays the overall process of the proposed algorithm.

The proposed segmentation model was designed to distinguish the cortical bone and marrow bone of the mandibular condyle. The model consisted of a segmentation sub-model and a classification sub-model, which determined whether the given axial images contained a condyle head or not.

**Figure 2:**
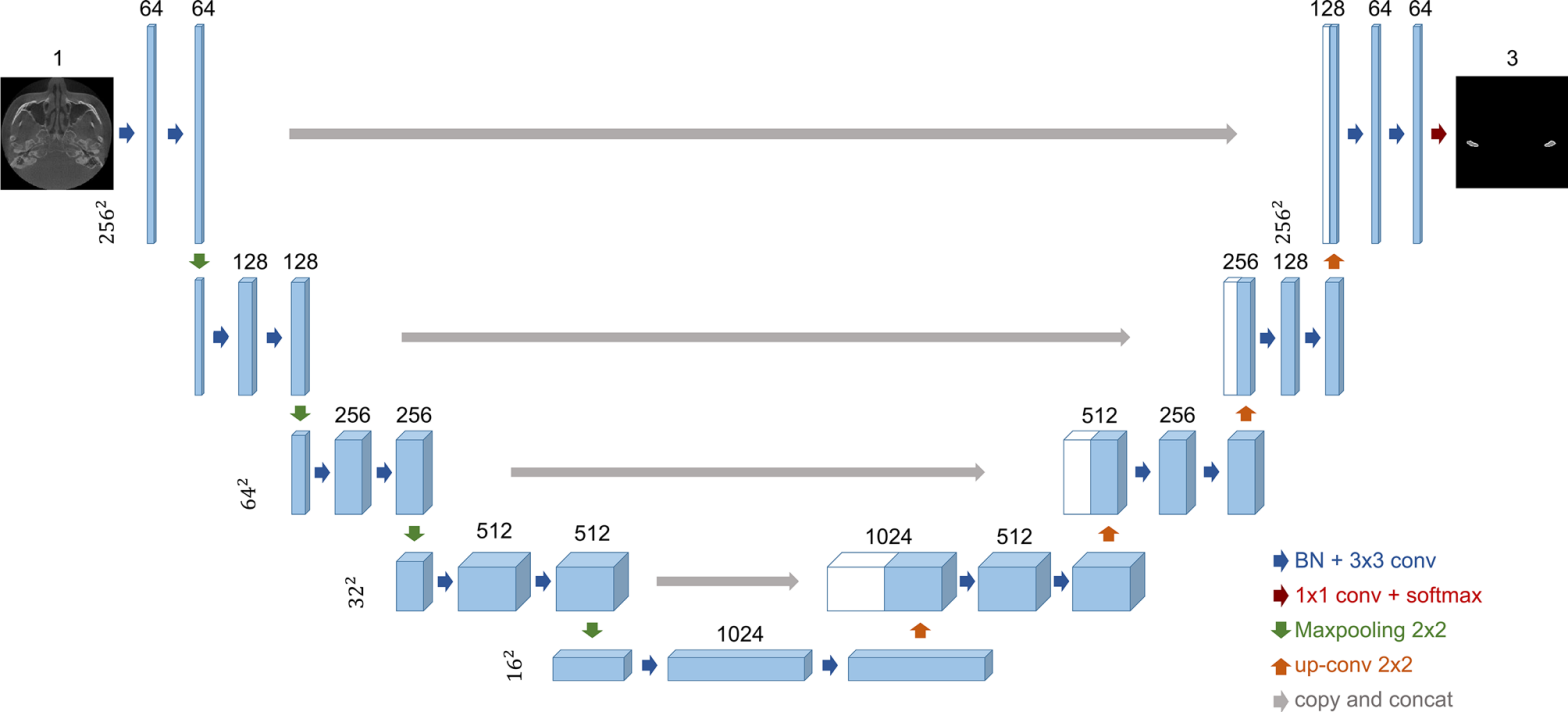
The proposed U-Net architecture used to segment the cortical and marrow parts of the condyle head. Unlike the original U-Net model, it uses batch normalization and concatenation without cropping.

First, we designed a U-Net-based model to segment the cortical and marrow parts of the condyle head as illustrated in Fig. 2.

The U-Net is a fully convolutional network^33^, which is one of the most successfully applied models for medical image segmentation tasks^34^. It consists of a contracting path and an expansive path. In the contracting path, the model extracts feature information from the images, but loses spatial information. To convey spatial information, shortcuts exist from the contracting path to the expansive path. Using this information, the expansive path restores the resolution of images for segmentation.

Every convolution layer used $3\times 3$ filters and ReLU except the output layer, which used $1\times 1$ filter and softmax. For downsampling and upsampling, we used $2\times 2$ maxpooling and upconvolution (deconvolution) with stride 2, respectively. Unlike the original U-Net, we used padding for each convolutional layer instead of cropping the feature map. Additionally, batch normalization^35^ was performed before every weighted layer to accelerate training. The detailed structure can be seen in Fig. 2. In our experience, a larger number of max-pooling layers and upconvolution layers did not improve the results, while a smaller number of those layers caused performance degradation in our tasks.

Our objective function was the sum of the cross-entropy and the dice loss^36^, which was defined as follows:

1 $${\text{DiceLoss}}\left( {p, \hat{p}} \right) = 1 - \frac{{\left( {2p \circ \hat{p}} \right)}}{{\left( {p \circ p + \hat{p} \circ \hat{p}} \right)}}$$

where $p, \hat{p}$ are vectors and the operator $\circ$ is the inner product. The second term is known as the dice coefficient. We used the following combined objective function:

2 $${\text{Loss}}\left( {p,{ }\hat{p}} \right) = {\text{DiceLoss}}\left( {p,{ }\hat{p}} \right) + {\text{CrossEntropy}}\left( {p,{ }\hat{p}} \right)$$

To classify the presence of the condyle head in the given axial slices, a classifier model was also developed. The classifier consisted of 2 convolutional layers with a kernel size of 5, 2 convolutional layers with a kernel size of 3, and a single linear layer. Each convolutional layer was followed by ReLU and a max pooling layer with a kernel size of 3. Therefore, it had little effect on the inference time.

To train the proposed U-Net and the classifier models, we used an Adam optimizer^37^ with a fixed momentum parameter (0.9, 0.999), a weight decay of $10^{ - 4}$, a batch size of .1 and a learning rate of $10^{ - 4}$. Hands-on tuning for the abovementioned hyperparameters was conducted to select suitable hyperparameters, but there was no significant difference, so fixed parameters were used. All axial images were used for training the classifier and only images with the condyle head were used for training the U-Net. To test the robustness of our algorithms, we repeated 50 experiments using Monte Carlo cross-validation^38^, a method of randomly subsampling data within the same dataset split ratio. In each experiment, a model was trained for 30 epochs. Among the weights for 30 epochs, the weight with the best evaluation metric (intersection over union, IoU) was selected. As a result, we obtained 50 different models, the performance of which is shown in Fig. 6.

**Figure 3:**
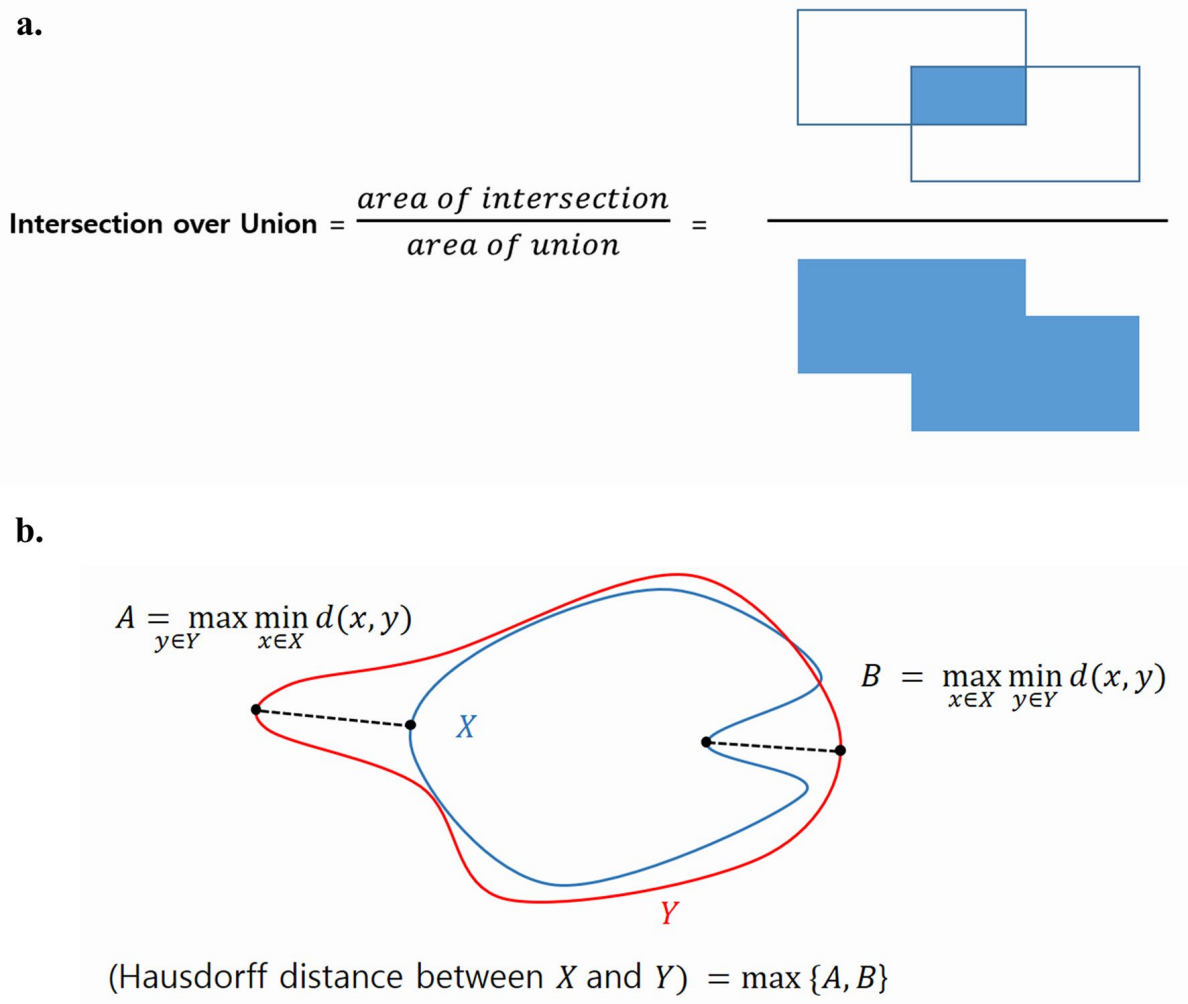
Graphical visualization of the intersection over union (**a**) and the Hausdorff distance (**b**). $d$ in (**b**) denotes the distance between two points.

The performance of the proposed CNN models was evaluated using the IoU and Hausdorff distance. The IoU is the most common evaluation metric used for benchmarking segmentation. It is defined as the area of overlap of the gold-standard label and the segmented label divided by the area of their union, and has values between 0 and 1 (Fig. 3a). As the prediction approximates the gold-standard, the IoU becomes closer to 1. In our experiment, the label contained two classes (cortical and marrow), so we measured the IoU of the cortical and marrow classes separately. The Hausdorff distance was used to evaluate the CNN architecture by measuring the distance of two points between the gold-standard label and the segmented label. More specifically, the Hausdorff distance is a measure of the distance between the most inconsistent parts of two sets (Fig. 3b).

### Proposed thickness estimation and visualization model

Based on the two-dimensional (2D) segmenting results, we rendered the 3D condyle head model using the marching cubes algorithm^39^. To obtain the coordinates of both surfaces, we used Canny edge detection^40^, which detects the edge of the image based on the intensity gradient and a given threshold. Because a label image consisted of only three values (cortical, marrow, and background), applying the Canny edge detection to labels enabled the accurate identification of the surface. Using the obtained coordinates of the surfaces, we measured the distance from each point of the cortical surface to the surface of the marrow bone. Of note, the thickness that we measured was the length of the line segment perpendicular to the surface of the marrow bone. To compare these results with the actual thickness, we applied the same method to the expert-labeled data and compared both results using the distribution graph of cortical bone thickness. Finally, the measured cortical bone thickness values were visualized using colors so that the automatic segmentation and thickness estimation results could be easily evaluated.

## Results

### Segmentation results

**Figure 4:**
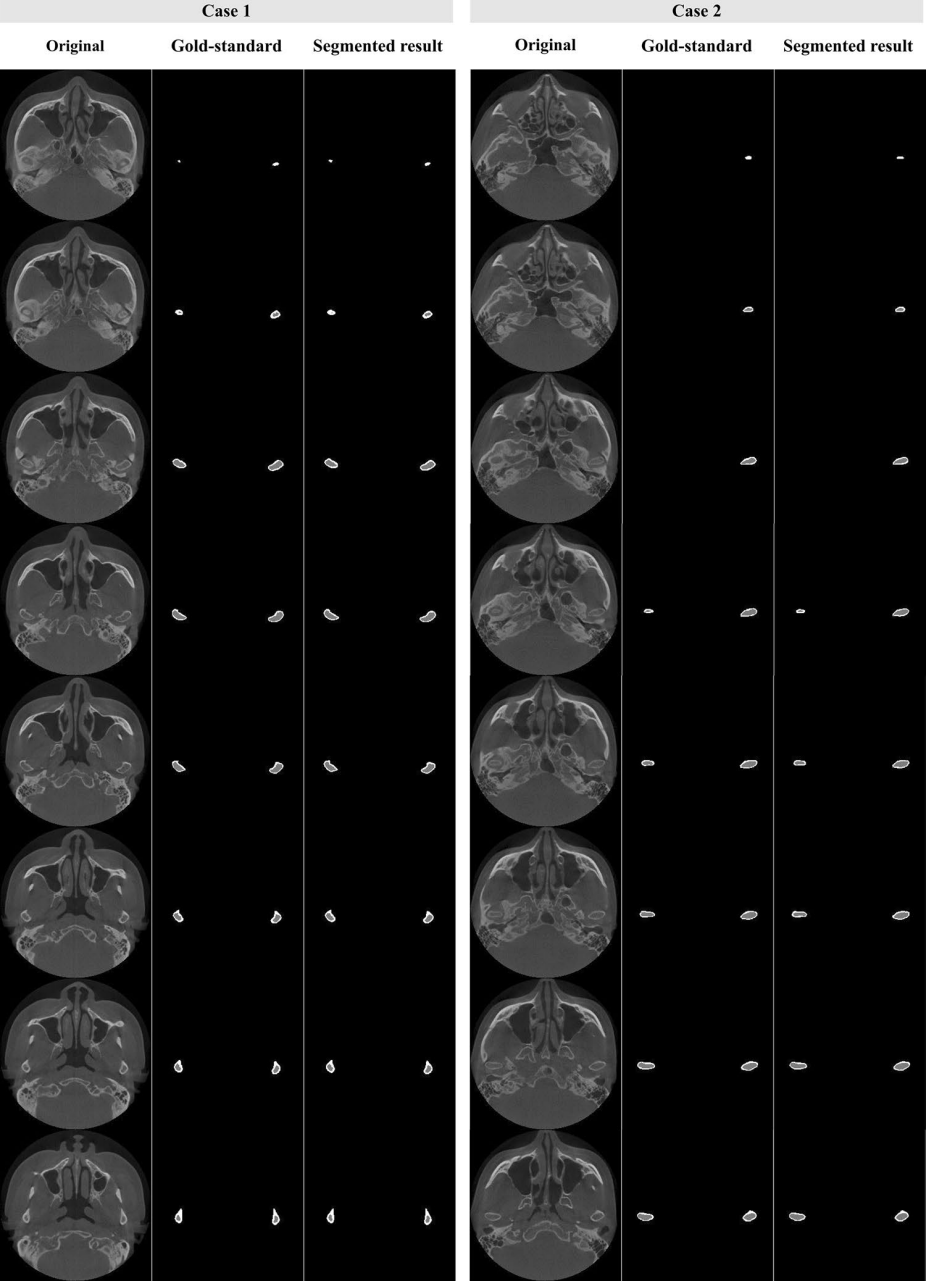
Examples of visual evaluation of the deep learning-based segmented model. The gold-standard is the manual labels annotated by an expert and the segmented result is the labels predicted by the proposed convolutional neural network algorithm.

**Figure 5:**
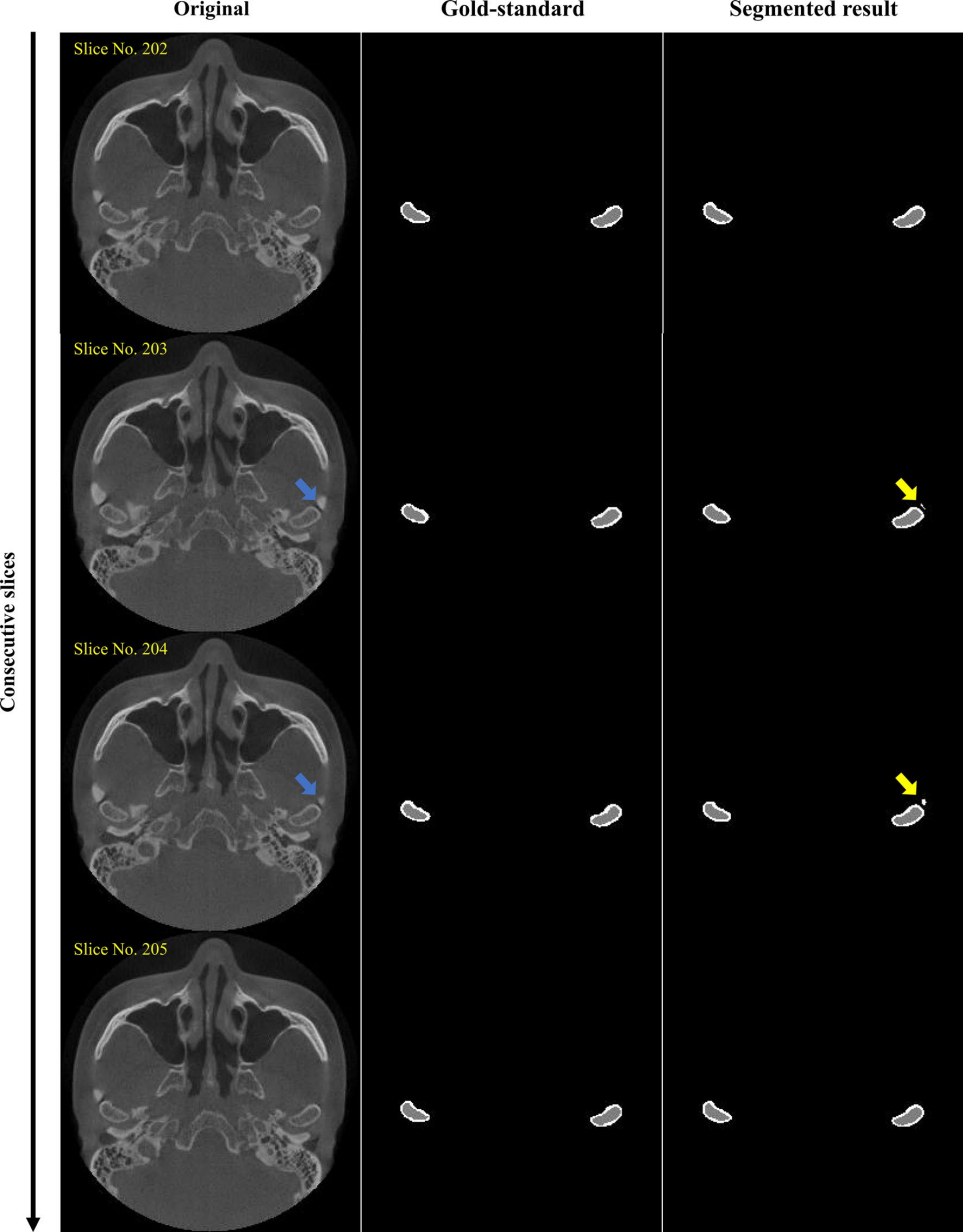
Examples of poorly segmented results obtained from the proposed segmentation model. From the consecutive slices, two poorly segmented slices were observed. The blue arrows indicate the surrounding anatomical structure of the condyle head, and the yellow arrows indicate the mis-segmented areas.

As shown in Fig. 4, we compared the original images, gold-standard labels, and segmented results obtained using our model. The proposed algorithm automatically selected only the condyle head from the whole head image, and then segmented the cortical bone and marrow bone within about 10 s. The oral radiologist who annotated the gold-standard labels performed a visual inspection of all segmented images, and confirmed that the proposed algorithm segmented the TMJ condyle head well, with results similar to the gold-standard images. However, some poorly segmented results can be seen in Fig. 5. In some images, the model misrecognized and segmented the surrounding anatomical structures.

**Figure 6:**
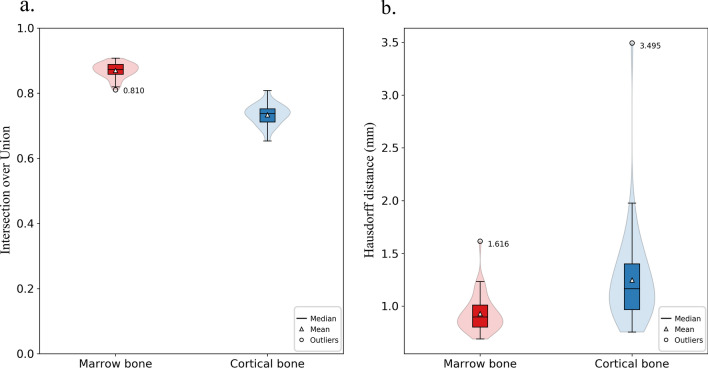
Box and violin plots of the performance metrics (IoU and the Hausdorff distance) obtained during cross-validation experiments. The black dots and their values represent outliers.

The objective performance of the proposed CNN algorithm was evaluated by the IoU and Hausdorff distance between the gold-standard labels and the segmented results. Figure 6 shows the results of the performance metrics for test cases over 50 cross-validation experiments. Overall, the CNN algorithm worked better with the marrow bone. The mean IoU value for the segmented marrow bone was 0.870 ± 0.023 (range: 0.810–0.908), while that for the cortical bone was 0.734 ± 0.032 (range: 0.653–0.808) (Fig. 6a). In Fig. 6b, the mean Hausdorff distances were 0.928 ± 0.166 mm (range: 0.689–1.616 mm) ﻿and 1.247 ± 0.430 mm (range: 0.755–3.495 mm) in the marrow and cortical bone, respectively. A few outliers led to greater mean Hausdorff distance values for both the marrow and cortical bone.

### Thickness estimation and visualization results

**Figure 7:**
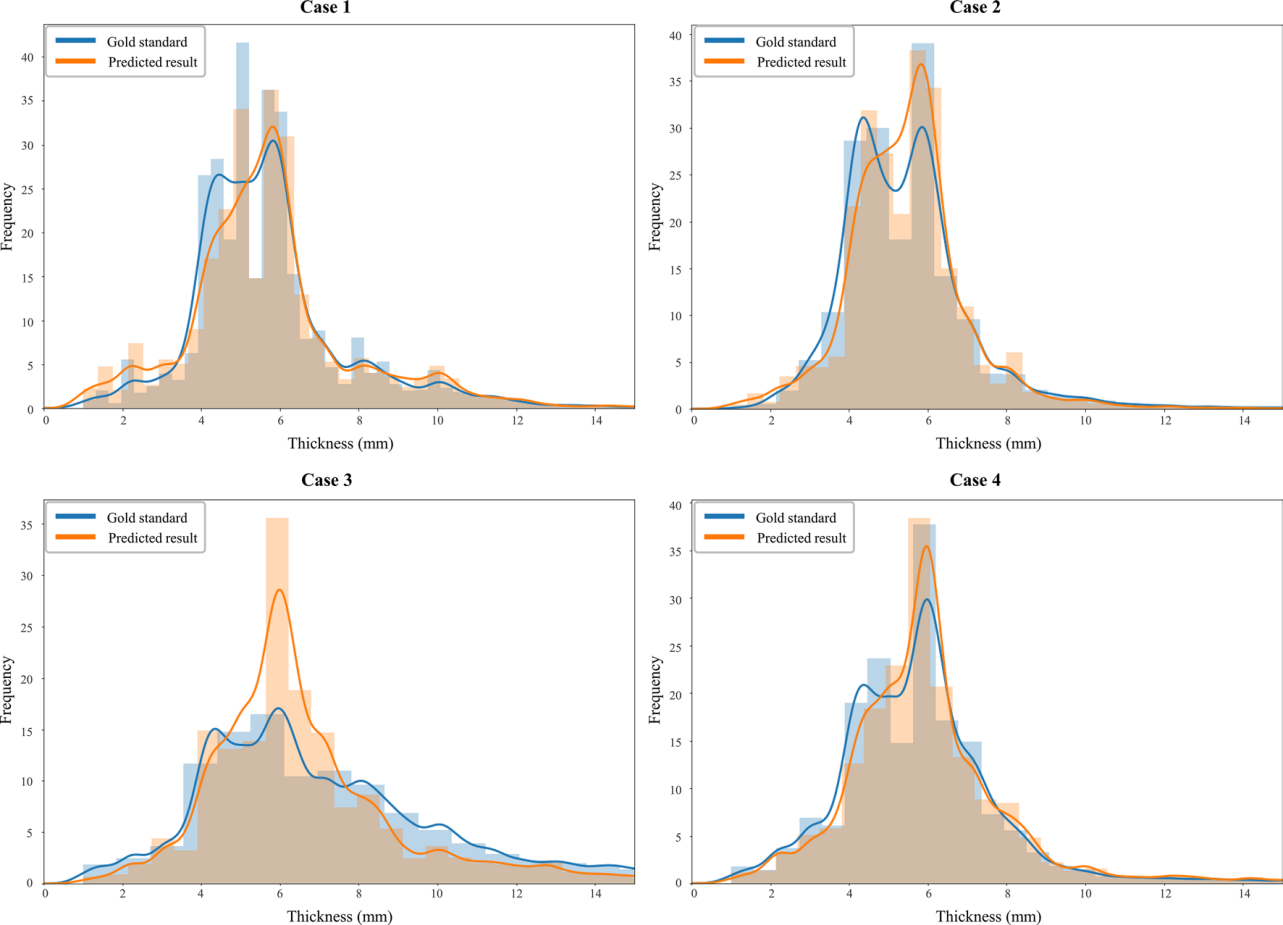
Distribution graphs of the cortical bone thickness from four randomly selected test cases over 50 experiments. The blue and orange curves were obtained from the gold-standard and segmentation results, respectively.

**Figure 8:**
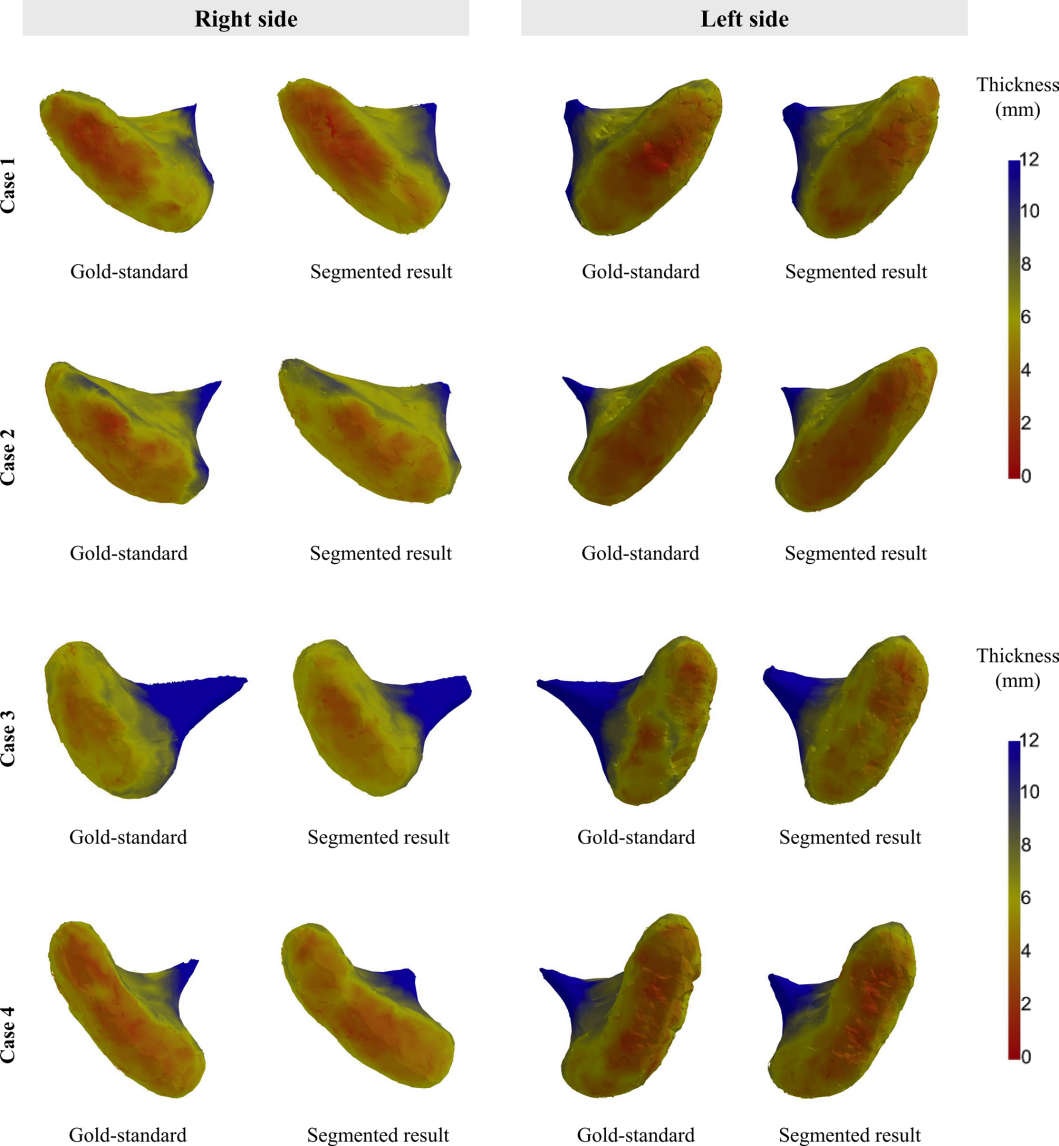
Visual comparison between the gold-standard and segmentation results by color mapping or cortical thickness on a three-dimensional temporomandibular condyle head surface.

Figures 7 and 8 present the distribution graphs of the cortical bone thickness and 3D rendered condyle head with a color map from four randomly selected test cases over the 50 cross-validation experiments. Overall, a similar trend can be seen in the gold-standard and the automatic measured thickness distributions, but the difference seems to be less for thin cortical bone (less than 4 mm thick) (Fig. 7). In Fig. 8, the color location and distribution of the condyle head as visualized by the expert-labeled images and the proposed algorithm were analogous. In case 3, a large difference from the gold-standard distribution in the 6-mm thickness range (Fig. 7) could be visualized at the center of the left condyle head (Fig. 8).

## Discussion

Increasingly many patients are suffering from TMD worldwide^41,42^. Bony changes such as sclerosis, flattening, bone erosion, or osteophyte formation are found in the mandibular condyle head in patients with osteoarthritis, which may indicate the progression of degenerative disease in TMD^5,43^. Considering that the pathological process manifests by thickening and remodeling of the TMJ condylar surface over a period of time^7^, separating specific bone structures and measuring their thickness would help clinicians assess degenerative changes.

In our implementation, we automatically classified the condyle head from CBCT images by merging an additional classification network that does not rely on human interventions. This means that there is no need for clinicians to manually select a target region, such as the condyle head, from the full image for segmentation. The proposed classifier model takes the same input as the U-Net and has a much simpler architecture, which has little effect on the inference time. Since the networks automatically classify the condyle head and its cortical bone from the full CBCT data, the algorithm can produce consistent and reliable predictions. Moreover, the total time required to produce a single CBCT segmentation is only 10–15 s (with an NVidia Titan V GPU). Therefore, this model can be used for statistical analyses incorporating high volumes of CBCT data, and in further studies, it will be possible to analyze enormous numbers of patient images in a short time.

Occasionally, the proposed classifier made non-consecutive predictions for a few images. The prediction for consecutive axial images should not oscillate between 0 and 1. To solve this problem, we considered using a 3D convolutional neural network, such as a V-Net. However, such models would require higher computational costs and a larger dataset to train. Instead of modifying the model architecture, we used the kernel filter to post-process the predicted binary vector. Using the uniform kernel, we picked images with values exceeding a fixed threshold. This simple processing led to satisfactory results.

The outstanding performance of our automatic segmentation model was demonstrated by a high IoU threshold (0.7) and mean distance errors of around 1 mm in both bone marrow and cortical bones during 50 repeated experiments. However, in the cortical bone, a large deviation was observed in the range of the Hausdorff distance from 0.755 to 3.495 mm. Considering that the distance value corresponding to 75% of the segmented results was 1.165 mm, an error exceeding the average value (1.247 mm) may be an outlier. Although the model worked properly for most data, the model intermittently misrecognized the surrounding structures of the TMJ condyle head in roughly 1 or 2 images of all axial slices. This phenomenon greatly increased the Hausdorff distance, but it mostly occurred around the condylar neck and did not affect the interpretation of the 3D-rendered color map of the condyle head. The robustness of the model for surrounding structures would seem to be a task for future improvement.

We propose a fully automated process from the input of a whole CBCT image of a subject including the condyle region to the output of a 3D color map of segmented cortical bone thickness. Although threshold-based segmentation has been widely used, difficulties have been reported in establishing a reliable threshold value due to the inherent limitations of CBCT as an imaging modality, such as non-homogeneous image quality caused by X-ray scattering^23,31,44,45^. Subsequent semi-automatic techniques of determining the threshold by an algorithm have also been proposed, but even partially manual work tasks are still influenced by the operator's experience^46,47^. We expect to be able to minimize the variance of segmentation outcomes by reducing operator-dependent issues using our proposed method.

CBCT is widely used by dental clinicians due to its high diagnostic quality with a relatively low dose and cost^48^, and oral radiologists consider it to be an effective tool for examining bony changes in the TMJ^49–51^. However, the complexity of multi-sectional image reconstruction for TMJ diagnosis leads to variability in pathologic findings and diagnostic discrepancies, which can be an obstacle to reliable CBCT interpretation for clinicians and even experienced radiologists^43,50,52^. In order to overcome these obstacles and provide a diagnostic tool that can be easily used by clinicians, we developed a novel automatic estimation and visualization method of cortical bone thickness in the mandibular condyle head.

The proposed method provides a 3D-rendered condyle head with color maps according to the measured thickness by stacking the resulting 2D slices that were obtained from our segmentation deep learning model. In general, 3D images reconstructed from CBCT are considered inadequate to examine bony changes, since threshold-based 3D rendering can reconstruct a limited area depending on its density^53^. In other words, once the cortical bone is slightly changed (as in early osteoarthritis), it is difficult to detect the lesion area with a 3D-rendered image. Our 3D color maps, reconstructed based on the segmentation of cortical bone, can visualize changes in thickness as the disease progresses, providing diagnostic information for the TMJ condyle head region undergoing bony changes. We expect that the use of 3D maps will allow clinicians to identify pathological locations that were previously undetectable through 2D slice analysis alone and to assess quantitative bone changes of the condyle surface.

This study used data taken from a single CBCT device. It is necessary to use additional data from various CBCT devices to improve the performance of the algorithm. In this study, only normal subjects’ CBCT data were used, and correlations between the cortical bone thickness results using the proposed CNN algorithm and subjects’ clinical symptoms were not analyzed. Applying the proposed algorithm to large patient data sets with information on their clinical status would contribute to developing a treatment plan and predicting the prognosis of each patient.

Nonetheless, to our knowledge, this method is the first proposal based on an automatic and consistent assessment of cortical bone using a deep learning method in the mandibular condyle head, and this model has the potential to provide objective diagnostic information on the condition of cortical bone in the condyle head.

## Conclusion

In this study, we propose a novel automated method to diagnose bony changes of the TMJ condyle head from CBCT images. The proposed algorithm consists of a combination of a modified U-Net model for segmentation and another CNN model to classify the target region. In addition, a 3D color map uses differences in color to denote the thickness of the cortical bone in the segmented output. Through this algorithm, the cortical and marrow bones in the TMJ condyle head were segmented with high accuracy, and the measured cortical thickness was visualized as a 3D-rendered color map. This algorithm is expected to contribute to the automated quantification of bone thickness changes in CBCT image interpretation.
